# Benefits of global financial reporting models for developing markets: The case of Romania

**DOI:** 10.1371/journal.pone.0207175

**Published:** 2018-11-21

**Authors:** Mihaela Ionascu, Ion Ionascu, Marian Sacarin, Mihaela Minu

**Affiliations:** Department of Accounting and Auditing, The Bucharest University of Economic Studies, Bucharest, Romania; Anglo-American University, CZECH REPUBLIC

## Abstract

The paper explores the benefits of global financial reporting models for developing countries, discussing the case of Romania, which, at the recommendations of the World Bank and the International Monetary Fund, exceeded the minimum requirements of the European Union, by imposing the full adoption of the International Financial Reporting Standards (IFRS) in individual financial statements of listed companies. Using regression analysis and decomposition techniques, the paper explores the evolution in value relevance of financial variables based on pre-(2009–2012) and post-(2014–2016) adoption samples, showing that after IFRS adoption financial information becomes significantly more relevant for equity valuations. We also provide empirical evidence showing that the degree of relevance for stock valuation, as well as the IFRS impact varies across types of firms. Overall, our findings tend to indicate the success of the financial reporting reform, which could be relevant for other jurisdictions facing similar decisions.

## Introduction

Investors base their decisions on a complex set of information of various kinds, yet their primary interest lies in estimating the outlook for cash inflows of an entity that drives its ability to pay future dividends. In order to assess an entity’s ability to generate positive future net cash flows, investors need information on the entity’s economic resources and claims, but also on the efficiency and effectiveness of its resources administration. All this information is mainly financial in nature, and the companies’ financial reports are a major source of information for investors as they provide “much of the financial information they need” [1]. Therefore, an efficiently operating capital market requires entities to provide quality financial information in order to support investment decisions.

As a result of the need to increase the quality and comparability of financial information available on capital markets around the world, we are currently witnessing an unprecedented process of standardization of financial information. This process is led by the International Accounting Standards Board (IASB), which is a private independent foundation aiming at developing a single set of high quality, globally accepted rules: International Financial Reporting Standards (IFRS).

By implementing IFRSs globally, the IASB aims mainly at achieving three major benefits [2], *i*.*e*. removing barriers to cross-border investments, increasing the quality of financial reporting and reducing the cost of capital. These beneficial effects are expected to occur with greater intensity for countries with less developed economies, financial markets or local financial reporting systems. That is because, in addition to the adoption of a global financial communication language that facilitates investors’ access to local markets, the implementation of high quality accounting standards (such as IFRS) for such countries improves the quality of financial information, ensures transparency, reduces information asymmetry and risk, being a prerequisite for attracting and reducing the cost of capital.

For this reason, international financing bodies (*e*.*g*. the World Bank and the International Monetary Fund) have conditioned, in many cases the provision of access to finance for such national jurisdictions, with the implementation of IFRS [3],[4],[5]. In these countries, macroeconomic effects are expected [2], which will eventually lead to economic growth and to bridging the gaps to developed economies [6].

In the European Union (EU), listed companies mandatorily apply IFRS in consolidated accounts since 2005, member states being allowed to extend the application of IFRS in individual financial statements. According to European regulations [7], IFRSs have been adopted at the EU level “to ensure a high degree of transparency and comparability of financial statements and hence an efficient functioning of the Community capital market”, as the provisions of the European Directives were found wanting in this respect.

In this context, it is important to investigate the group of emerging countries in the EU, as they are expected to continue the process of European integration and to reduce the economic and institutional gaps to the advanced EU member states, because, as noted above, IFRS adoption is expected to have a much more significant effect in less developed countries. Therefore, the purpose of this paper is to investigate the consequences of IFRS adoption in one of the EU’s emergent markets–the Romanian market, a country, which recently went beyond the EU standard policy and adopted IFRS in individual accounts of listed companies as part of its obligations assumed within the World Bank and International Monetary Fund agreements.

As the EU aims at a more “*efficient functioning*” of the capital market [7], this paper examines the extent to which IFRS financial information is better incorporated within the Romanian stock market pricing. In other words, the paper investigates whether the adoption of full IFRS in individual accounts of Romanian listed companies manages to increase investors’ confidence in the financial information provided by these companies, reflected by their increase in ***value relevance***.

According to IFRS, ***relevance*** is one of the fundamental qualitative characteristic of financial information–which assumes it making a difference in investors’ decisions, having predictive value, confirmatory value for previous evaluations or both [1]. Consequently, the adoption of the high quality international reporting standards is expected to lead to increased confidence of investors in financial data–perceived as more trustworthy information, which should result in funds suppliers relying more on IFRS compliant information when making investment decisions.

## IFRS adoption in Romania

In Romania, the IFRS implementation has been performed gradually starting in 1999–2001, with the purpose of providing transparency by increasing the quality and comparability of financial information that could attract primarily foreign investments [8]. The passage to IFRS was done progressively in three stages (Table 1). In the first one, Romanian accounting system was harmonized with the IFRS principles, whereas in the last two phases, the aim was to achieve conformity with IFRS, initially in consolidated accounts, and subsequently in individual financial statements of listed Romanian companies.

**Table 1:** Mandatory IFRS adoption in Romania.

**1999–2005**	**Harmonization phase** Regulations harmonized with both the EU accounting directives and IFRS for individual financial statements
**2006–2011**	**Conformity phase for consolidated accounts** Consolidated accounts of listed companies in compliance with IFRS EU accounting directives in individual financial statements IFRS allowed in individual accounts on a voluntary bases, yet not valid in relation with state institutions
**2012-present**	**Conformity phase for individual accounts** Both individual and consolidated accounts of listed companies in compliance with IFRS

IFRSs were first introduced in Romanian in 1999–2001 at the request of international funding bodies (World Bank and International Monetary Fund) that assisted Romania in the transition to the market economy after the fall of communism. The adoption of IFRS has been included among the conditionalities of the financing agreements aimed at creating an attractive environment for foreign investments and privatization. Since Romania had a French-inspired system at that time, the switch to IFRS meant a major shift in accounting philosophy, which was expected to be challenging for the accounting profession and businesses alike. For this particular reason, but also due to Romania’s objective of entering the EU, the decision was not for full IFRS adoption, but only *harmonization* with the international accounting standards, regulations being issued in 2001 (after an initial experiment on 13 companies in 1999) for the *harmonization of Romanian accounting with both the EU Directives and IFRS* [8].

The harmonization phase meant only a limited application of IFRS, with individual financial statements being far from full IFRS compliant, as, in all probability, intended by the international funds suppliers. Given the Romania’s entry into the EU in 2007, the IFRS application policy has changed in order to meet EU’s mandatory requirements, which demanded *full compliance with IFRS for consolidated financial statements* of listed companies, and the application of the Fourth Directive in individual accounts. In this second stage, public interest companies were allowed to prepare individual accounts in accordance with IFRS, but only for their own information needs and only as a second set of financial statements, which was not officially recognized in relation with state institutions [9]. For banks, preparing a second set of accounts in compliance with IFRS was mandatory since 2009 [6].

However, EU allows member states to extend the application of IFRS beyond consolidated accounts of listed companies [7], which made the third phase of the IFRS adoption possible. In 2012, Romania issued regulations that imposed mandatory *full IFRS compliance for individual accounts* of listed companies starting in 2012, explicitly mentioning the “recommendations of international organizations, namely the World Bank and the International Monetary Fund” as one of the incentives for their decision [10]. The year 2012 was regarded as a *transitional year*, companies still drafting two sets of financial statements (IFRS compliant accounts being obtained by restating those prepared in accordance with EU Directives). In addition to companies listed on a regulated market and banks, Romania is also considering extending the application of IFRS in individual accounts of unlisted state owned firms, insurance companies or other financial entities under the supervision of the Romanian Financial Supervisory Authority, such as brokerage or assets management entities.

## The Bucharest Stock Exchange

The Bucharest Stock Exchange (BSE) is a very young market as, although it was originally established in 1882, it only reopened in 1995, after being closed for approximately 50 years during the communist regime.

In 2005, BSE absorbed the RASDAQ market (RASDAQ Romanian Association of Securities Dealers-Automated Quotation), a market established in 1996 for trading securities of more than 6,000 former state-owned companies entered into the mass privatization program, which failed to meet the listing requirements of BSE, and constituting a separated component of the market. This made BSE the only stock market in the country. The number of companies listed on the RASDAQ segment of BSE continuously declined, and in 2014, the Romanian parliament decided on its dissolution. The 900 firms still listed on RASDAQ at that time [11] were offered a choice to promote to the regulated segment of the market or list on the new “equities segment of BSE’s Alternative trading system (ATS)” [12]–the AeRO market—established in 2015 for start-ups and SMEs.

In 2017, there were 84 companies listed on the regulated market (23 companies in the Premium tier and 61 in the Standard tier), and 274 companies listed on the AeRO segment of BSE [13]. Listing requirements for market segments are presented in Table 2.

**Table 2:** Listing criteria for BSE market segments.

**Regulated market** *Standard Tier*	**Listing requirements**: Joint-stock companies; Share capital or anticipated market capitalization of € 1 million; Minimum 25% free-float; Minimum 3 years record of financial reporting.
*Premium Tier*	**Additional listing criteria for Premium Tier**
	Stock conditions: registered with the Financial Supervisory Authority; freely transferable; fully paid in; issued in a dematerialized form; same class; minimum free-float of € 40 million. Issuer conditions: fee payment; permanently and fully informing the public about both the important events and the decisions that may affect the price of the shares; pay dividends without privileges and without discrimination based on fair and equitable criteria.
**AeRO market**	**Listing requirements**: Joint-stock companies; Share capital or anticipated market capitalization of € 0.250 million; Minimum 10% free-float or minimum 30 stockholders.

**Source**: Selected from data reported by BSE: http://bvb.ro/ForCompanies/MainMarket/IssuingShares, http://bvb.ro/ForCompanies/AeroMarket/IssuingShares, http://www.bvb.ro/Juridic/files/Cod%20BVB%20op%2002082016.pdf

The Romanian stock market is one of the smallest in region, both in terms of its market capitalization and its percentage of market capitalization in GDP (Tables 3 and 4). Romania’s accession to the European Union in 2007 has positively influenced the Romanian capital market, due to the expectations of foreign investors, when the market registered the most important traded values, as well as the highest increases in stock indices [12]. However, the 2007–2008 financial crises cancelled this significant expansion of the market, in 2008 BSE losing two thirds of its market capitalization (Table 3).

**Table 3:** Market capitalization of Central and Eastern European stock exchanges (billion €).

Stock Exchange/Year	2006	2007	2008	2009	2010	2011	2012	2013	2014	2015	2016
Athens Exchange	157.34	181.23	65.27	78.9	50.38	26.02	34.04	59.94	45.58	37.55	35.3
**Bucharest Stock Exchange**	**18.86**	**21.52**	**6.47**	**8.4**	**9.78**	**10.82**	**12.09**	**17.83**	**18.39**	**16.97**	**16.81**
Bulgarian Stock Exchange–Sofia	7.83	14.82	6.37	6.03	5.5	6.3	5.03	5.09	4.99	4.39	4.95
CEESEG Budapest Stock Exchange	31.37	31.48	13.31	20.71	20.71	14.63	15.74	14.36	12.01	16.19	21.27
CEESEG Ljubljana Stock Exchange	11.51	19.7	8.47	8.46	6.99	4.87	4.91	5.17	6.21	5.52	4.99
CEESEG Prague Stock Exchange	34.69	47.99	29.62	31.27	31.92	29.2	28.19	21.99	22.84	23.54	22.19
CEESEG Vienna Stock Exchange	146.2	161.73	54.75	77.24	93.94	65.68	80.43	85.39	79.99	87.93	95.2
Warsaw Stock Exchange	112.83	144.73	67.91	105.16	141.92	107.48	134.76	148.68	139.07	126.02	130.98

**Source**: Federation of European Securities Exchanges, http://www.fese.eu/statistics-market-research/historical-data

**Table 4:** Market capitalization of Central and Eastern European stock exchanges as percentage of GDP.

Country/Year	2006	2007	2008	2009	2010	2011	2012	2013	2014	2015	2016
Austria	54.86	57.28	18.75	26.99	31.88	21.28	25.36	26.47	24.21	25.87	27.25
Bulgaria	28.78	45.67	17.12	16.16	14.39	15.26	11.99	12.12	11.67	9.69	10.45
Czech Republic	28.03	34.77	18.40	21.08	20.41	17.80	17.46	13.94	14.58	14.10	12.72
Greece	72.22	77.88	26.97	33.22	22.29	12.57	17.80	33.18	25.62	21.37	20.07
Hungary	34.32	30.96	12.37	22.08	21.06	14.51	15.89	14.15	11.44	14.76	18.92
Poland	41.09	46.11	18.55	33.16	39.23	28.27	34.61	37.67	33.84	29.30	30.87
**Romania**	**19.16**	**17.16**	**4.54**	**6.98**	**7.72**	**8.12**	**9.06**	**12.36**	**12.23**	**10.61**	**9.91**
Slovenia	36.47	56.04	22.32	23.39	19.28	13.20	13.64	14.39	16.63	14.31	12.55

**Source**: Computed based on Table 3 data and GDP figures reported by Eurostat, http://ec.europa.eu/eurostat/tgm/refreshTableAction.do?tab=table&plugin=1&pcode=tec00001&language=en

From 2009, BSE has registered a steady increase, by the end of 2014 market capitalization reaching its pre-EU-accession level. Due to the good performance of the market, in 2016 rating agencies (FTSE Russell) included Romania on the list of countries that have the potential to achieve the *emerging market status* in a short or medium term [14].

The stage of development of a capital market is also given by its level of efficiency, *i*.*e*. the extent to which prices incorporate relevant information available. Most of the empirical evidence seems to support a *weak form of efficiency* for the Romanian capital market (*e*.*g*. [15], [16], [17], [18]), although there are also studies concluding that BSE is inefficient (*e*.*g*. [19], [20]).

## Previous research on the value relevance of IFRS

In the literature, IFRS are considered a set of high quality accounting standards [21], and their application is expected to increase the quality of financial reporting, hence, its usefulness for capital suppliers. The impact of IFRS adoption on the quality of financial information can be measured either by focusing on the changes it induces onto the characteristics of financial reporting, or by means of the actual impact of the information compliant with IFRS on investment decisions. The latter approach assumes that the impact of IFRS on equity markets can be assessed by means of investigating a possible increase in the ***value relevance*** of accounting information, that is, by assessing the extent to which financial reporting comprises the relevant information that determine the companies’ value [22], [23] [24], [25]. That is because, if financial information is relevant, it is included in the market pricing mechanism. *Value relevance* is construed as a significant correlation between financial information and market ***prices***, ***price changes*** or ***returns***, the power of financial variables in explaining market variables giving the magnitude of value relevance [22], [24].

Although there is not yet a consensus reached in the literature, most empirical results show that IFRS adoption improves the quality of financial information, which becomes more correlated with market variables. Thus, Niskanen *et al*. [26], after analyzing the information content of accounting data of Finnish listed companies, have found that between 1984 and 1992 earnings figures computed based on a voluntarily IFRS reconciliation were more value relevant that the ones based on Finnish accounting standards. Bartov *et al*. [27] compared the results of German companies that have applied local accounting standards and those that voluntarily applied IFRS for the period 1998–2000 and concluded that the results under IFRS are more value relevant than those computed under German accounting regulations.

Jermakowicz *et al*. [28] analyzed the benefits of voluntary adoption of IFRS by German companies that were included in the composition of DAX 30 index, noting that, during the period 1995–2004, the adoption of IFRS has increased the relevance of the financial information related to companies’ performance.

Barth *et al*. [22] conducted a research on a large sample of firms from 21 countries that have adopted IFRS between 1994 and 2003 and pointed out that companies applying IFRS feature a higher quality of financial reporting than the others, as they experienced an increase in value relevance together with a decrease in earnings management and an increase in the timely recognition of losses.

In France, Lenormand and Touchais [29] found that, at the end of 2004, the value relevance of financial reporting for 160 French companies whose securities underlie the SBF 250 stock index, increased with mandatory adoption of IFRS. Iatridis [30] also states that, in the UK, British transition to IFRS accounting standards has increased the value relevance of financial information.

For Italian companies, tests conducted by Paglietti [31] on value relevance of accounting data show that the mandatory application of IFRS has improved the quality of financial data used by investors. Also in Greece, after the mandatory IFRS adoption, since 2005, the value relevance of accounting information of listed companies has been reported to increase [32], [33], [34].

In the case of countries with less developed financial markets, there are studies documenting an increase in the value relevance of accounting information, with the shift from local standards to IFRS. For example, Karğin [35] found that for the period 2005–2010, after IFRS adoption, the value relevance of financial information increased for Turkish firms, accounting figures being more correlated with market values. Also, for the Chinese financial market, the study by Liu and Liu [36] showed that accounting figures reported under IFRS are more relevant than those reported under Chinese accounting principles.

However, there are studies that do not confirm an increase in the quality of financial reporting after the adoption of IFRS. For example, for Swiss companies, which have applied both IFRS and Swiss accounting standards, Babalyan [37] suggests that the application of IFRS does not necessarily involve an increase in the value relevance of the IFRS based financial information compared to the ones drafted under Swiss accounting standards.

Also, Hung and Subramanyam [38] observed that in the case of German companies which voluntarily applied IFRS for the first time in the period 1998–2002, net income and equity values determined under IFRS were not more relevant than those determined based on German accounting regulations. Likewise, Van Tendeloo and Vanstraelen [39], based on a research conducted for the period 1999–2001, concluded that German quoted companies that voluntarily adopted IFRS did not experience a reduction in earnings management, and, consequently, an increase in the quality of accounting information. Also in Germany, Pannanen and Lin [40] observed a decrease in the quality of accounting information for German companies after the mandatory application of IFRS

At the same time, for other European countries, IFRS adoption has not been documented to improve the value relevance of financial information. For instance, Jeanjean and Stolowy [41] observed that the mandatory transition to IFRS has not resulted in a major improvement in the quality of net income figures, as long as earnings management increased in France and remained constant in the UK. In addition, Callao *et al*. [42] found that mandatory application of IFRS has had a negative effect on the value relevance of financial reporting in Spain and in the UK.

Paananen [43] has also observed a lack of the expected increase in the quality of financial reporting for Swedish companies in the following two years after the mandatory adoption of IFRS in 2005. Contrariwise, [43] documented a decrease in the quality of financial reporting in terms of value relevance, earnings management and timely loss recognition. Dobija and Klimczak [44] explored the financial reporting in Poland and concluded that financial market efficiency and relevance of accounting information have not improved with the adoption of IFRS starting in 2005.

The notable research that investigated the value relevance of financial information in Romania, and the impact of IFRS on value relevance was made by Filip and Raffournier [24]. Their study covered the *harmonization with IFRS phase* (1998–2004), the phase that aimed at drawing Romanian accounting nearer to the international regulations, the harmonized financial reporting being, however, far from full IFRS compliant. Based on a return model, [24] documented that financial information in Romania had a relatively high value relevance compared to more developed markets, which was explained by the lack of transparency of the Romanian environment, the information provided by listed companies being among the few available to investors. In addition, their results indicated that the value relevance of financial reporting harmonized with IFRS had slightly increased compared to that of financial reporting compliant with local accounting standards.

However, Filip and Raffournier [24] failed to acknowledge that these results were largely affected by an anomalous result, *i*.*e*. a *significant negative* correlation between changes in earnings and market returns (t statistic −5.588 for changes in earnings added to 7.358 reported for earnings). This particular anomaly led them to question the premises of the return model they used, concluding–based on further tests—that the model’s hypothesis are not suitable for the Romanian environment, the low efficient capital market failing to timely incorporate financial information related to the variation in earnings per share. In particular, they reject the ‘price lead earnings’ hypothesis for the Romanian market, proposing a lag of six months for the return window initially used, computing both 12 and 18 months returns (July N–June N+1, and January N–June N+1, instead of January N—December N originally used) in order to allow prices to incorporate accounting information. The value relevance decreased drastically, from around 20% to approximately 7% and 5% for 6 months lagged 12 months and 18 months returns, respectively. Yet no follow up analysis was performed in order to investigate the IFRS harmonization impact based on the recalibrated return model, which renders Filip and Raffournier’s [24] results inconclusive.

Other studies addressing value relevance in Romania are scarce, relying on different and sometimes unreliable methodological approaches, a common feature being data handpicked directly from financial statements, without adjustments for stock splits or stock dividends. Although these studies employ rather basic methodologies, they still offer a glimpse onto the magnitude of the value relevance of financial data for the Romanian market.

Jianu *et al*. [45] focus on the *EU directives phase* (2005–2008) in order to investigate the effect of, allegedly, more”investor-oriented” regulations that introduce substance over form principle and *de jure* disconnection of accounting from taxation.

However, their research strategy and narrative are problematic, as, on the one hand, the switch from *regulations harmonized with IFRS* to *EU directives compliant regulations* was hardly an improvement, and, on the other hand, the period investigated was too eventful to allow for emphasising any potential impact of accounting regulations on value relevance. The year 2007 was the year Romania entered the EU, which converted into massive increases in capital investments, the significant upraise of the BSE being, subsequently, cut short in 2008, when the financial crisis hit the Romanian market, BSE loosing approximately 70% of its market capitalisation.

Jianu *et al*. [45] used both return and price models, with prices at the beginning of the year, and based their analysis on manufacturing listed companies only, which limits the generality of their results. Adopting a strategy similar to [24] in terms of prices dates, the return model also featured a significant negative coefficient for changes in earnings, preventing them from interpreting the effect of both independent variables (earnings and changes in earnings). The price model including earnings per share and book value of equity was found to provide more reliable results–with adjusted R squares increasing from 0.368 in 2005 to 0.706 in 2008 with a peak of 0.946 registered in 2007. The apparent increase in value relevance (significance of the increase not tested), was alleged as due to “improvement in accounting rules”. Yet, for the reasons stated above, such inference is hard to sustain.

Other studies, such as Mironiuc *et al*. [46], report questionable results (e.g. extremely high R squares for net income, with values approaching 100% for 2011–2013, based on a price model with a 6 months lag), probably due to their methodological tactics.

In this context, this paper attempts to provide more conclusive and more comprehensive evidence on the value relevance of financial information in Romania, and the impact of IFRS on its value relevance, focusing on a relatively stable period of the Romanian capital market (2009–2016), that could permit stressing out the effect of increasing the quality of financial reporting regulations.

## Research methodology

To compare the value relevance of financial information before and after IFRS adoption, we focus on the years 2009–2012 (before) and 2014–2016 (after the adoption of IFRS). The year 2013 was excluded as a transitional year in which the companies have disclosed financial information both under the European directives, and IFRS, the latter being obtained by restatement. Empirical results reported in the literature are inconclusive for the year in which IFRSs are introduced, the change in the characteristics of the reporting system probably confusing the users in a first stance, the actual effects on the market being felt with some delay. For readability purposes, year 2013 has not been emphasised in the figures reporting our findings.

**Fig 1:**
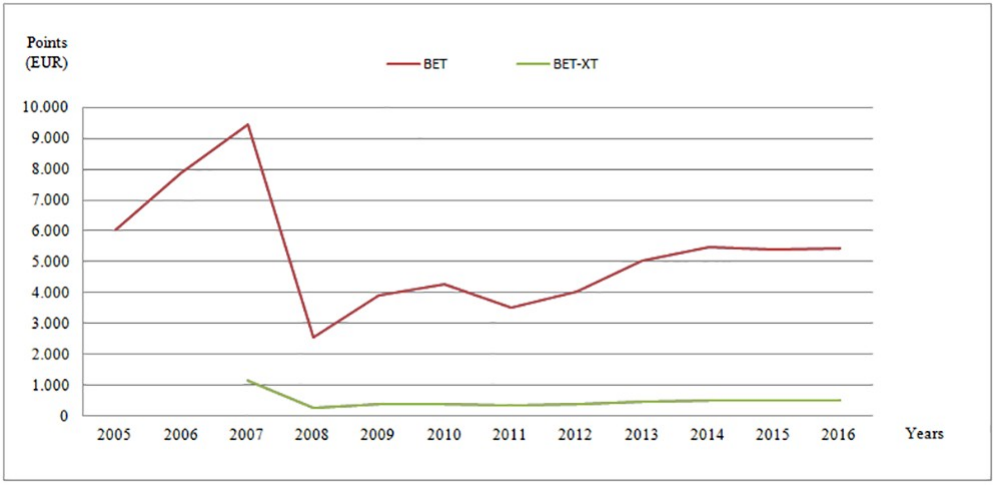
Romanian stock market evolution during 2005–2016. BET (Bucharest Exchange Trading) is the benchmark index for the performance of the BSE’s regulated market segment. Includes most traded listed companies, excluding financial investment companies. BET-XT (Bucharest Exchange Trading Extended) follows the first 25 most traded companies, including financial investment ones, listed on the regulated market segment of BSE. Source: http://bvb.ro/FinancialInstruments/Indices/IndicesProfiles.

Although full compliance with accounting European Directives was required for individual financial statements since 2006, the period before IFRS adoption excludes the years 2007 and 2008, as marked by significant events that could affect value relevance of accounting data (2007: EU accession year, 2008: major financial crisis impact on the Romanian market, BSE losing two thirds of its market capitalisation). The period 2009–2016 is relatively uneventful, BSE’s market indicators displaying a rather stable performance, which provides for a good opportunity to discuss the impact of switching to IFRS in individual financial statements (Fig 1).

Between the two alternative approaches used to determine value relevance, we opted for examining ***price levels*** and not price changes or returns. We decided on price models following Barth *et al*. [25], who argue that these models are appropriate if the interest lies on “determining what is reflected in firm value”, and not changes in prices or return models, which are useful in “determining whether the accounting amount is timely”. Barth *et al*. [25] uses the case of assets revaluations to show that value relevant, yet not timely, financial figures can be wrongly inferred as being valuation irrelevant when employing return models. As shown for the Romanian stock market, return models have already produced anomalous results [24], [45].

We also follow Barth *et al*. [47] in an attempt to offer a more comprehensive view on the value relevance of financial information, focusing not only on ***net income*** and ***book value of equity***, which are common figures addressed by the literature [47], [23], but also on other accounting amounts which are presumed to be linked to stock valuation, *i*.*e*. ***sales***, ***total assets***, ***intangibles***, ***cash holdings*** and ***dividends***, and the impact of IFRS on their value relevance. We limit the analysis to the above indicators due to data availability.

We first estimate value relevance based on net income and book value of equity (Eq (1) referred to as Basic Model):

$$P_{i}=\alpha_{0}+\alpha_{1}{NI}_{i}+\alpha_{2}{BVE}_{i}+\varepsilon_{i}$$ (1)

Where,

**Table:** 

*P*_*i*_	market price measured three months after financial year-end (*TF*.*PriceCloseQ1*);
*NI*_*i*_	earnings per share (*TF*.*NetIncome deflated by TF*.*CommonSharesOutstanding*);
*BVE*_*i*_	book value of equity per share (*TF*.*TotalCommonEquity deflated by TF*.*CommonSharesOutstanding*).

Year subscripts omitted for convenience.

Company indexed by subscript *i*.

Definition from Thomson Financial (TF) in parenthesis.

We use stock market prices with a three months lag in order to allow prices to incorporate financial data, which is consistent with relevant literature (e.g. [47], [48], [49]). Value relevance is measured by the coefficient of determination (R^2^).

We are also interested in the value relevance of each individual financial variable addressed by this study, as well as in their combined contribution to stock valuation. Therefore, we also estimate Eqs (2) and (3):

$$P_{i}=\alpha_{0}+\alpha_{1}{VAR}_{i}+\varepsilon_{i}$$ (2)

$$P_{i}=\alpha_{0}+\alpha_{1}{NI}_{i}+\alpha_{2}{BVE}_{i}+\alpha_{3}{SALES}_{i}+\alpha_{4}{TA}_{i}+\alpha_{5}{INTAN}_{i}+\alpha_{6}{CASH}_{i}+\alpha_{7}{DIV}_{i}+\varepsilon_{i}$$ (3)

Where,

**Table:** 

*SALES*	Sales (*TF*.*Sales deflated by TF*.*CommonSharesOutstanding*);
*TA*	Total assets (*TF*.*TotalAssets deflated by TF*.*CommonSharesOutstanding*);
*INTAN*	Intangibles (*TF*.*Intangibles deflated by TF*.*CommonSharesOutstanding*);
*CASH*	Cash and cash equivalents (*TF*.*CashAndSTInvestments deflated by TF*.*CommonSharesOutstanding*);
*DIV*	Dividends (*TF*.*DividendsPerShare*, *except for the year 2014*, *for which data was collected manually from BSE website*: http://www.bvb.ro/FinancialInstruments/Markets/Shares);
*VAR*	Each of the eight accounting variables.

All variables measured as per share data.

Definition from Thomson Financial (TF) in parenthesis.

The source for data collection was Thomson Financial database. Following Barth *et al*. [47] we imposed minimum requirements to our sample, *i*.*e*. we require companies to have non-missing net income, sales, book value of equity, total assets, share prices, and total shares outstanding. All other missing amounts we set to zero. Most of the missing amounts were found among dividends per share, in many cases reflecting real circumstances of Romanian companies not distributing dividends. However, this procedure severely limited the variability of dividends data for the year 2014, for which we collected data manually from BSE website [13].

Out of the 83 companies listed on BSE (27.7% on the Premium tier) as of December 31, 2016 (Table 5), missing data reduced our sample size to an average of 58 firm-observations per year, our full sample consisting of 406 firm-year observation, of which 26.6% loss firms, 16.3% financial companies and 29.8% companies listed on the Premium tier.

**Table 5:** Listed companies by industry.

Industry ^a)^	Frequencies	Percentages
Accommodation and food service activities	4	4.82
Construction	5	6.02
Electricity, gas, steam and air conditioning supply	3	3.62
Financial and insurance activities	12	14.46
Human health and social work activities	1	1.20
Manufacturing	45	54.22
Mining and quarrying	4	4.82
Professional, scientific and technical activities	1	1.20
Transportation and storage	4	4.82
Wholesale and retail trade	4	4.82
**Total**	**83**	**100**

^**a)**^ Romanian industry classification codes (CAEN—Classification of National Economic Activities) in compliance with United Nations’ ISIC Rev. 4 (International Standard of Industrial Classification of All Economic Activities) and Eurostat’ NACE Rev. 2 (Statistical classification of economic activities in the European Community)

Outliers were identified based on Tukey’s model with a 2.2 multiplier [50]. All data was winsorized by the nearest unsuspected value by year. Data was analysed with the functions of SPSS and R [51] (including the package Relaimpo [52]).

## Results and discussion

### Descriptive statistics and correlation analysis

Table 6 reports descriptive statistics, which further exposes the small size of the Romanian stock market when compared to developed ones, *e*.*g*. a mean stock price of RON 1.334 (approximately USD 0.31 based on the exchange rate at the end of 2016, compared to USD 18.89 reported for the American market [47].

**Table 6:** Descriptive statistics.

Variables	Mean	Median	Std. Deviation
***PRICE***	1.334	0.445	1.838
***NI***	0.058	0.019	0.173
***SALES***	2.587	0.564	3.657
***BVE***	1.933	0.618	2.717
***TA***	5.006	1.404	7.131
***INAN***	0.009	0.002	0.013
***CASH***	0.219	0.038	0.348
***DIV***	0.017	0.000	0.039

Table 7 provides information on the correlation between variables. All accounting variables selected are correlated with equity prices, which gives an indication of their value relevance. Total assets and book value of equity have the largest correlation coefficient (0.802 and 0.797), followed by net income with 0.632. The results are consistent with previous studies, book value of equity being reported as more value relevant than net income [47], [23], [49]. However, a distinctive feature is the position of total assets, which normally ranks below book value of equity and net income [47]. Total assets also feature large correlation coefficients in relation with sales (0.836) and book value of equity (0.834), which raises collinearity concerns.

**Table 7:** Variables correlation matrix.

**Variables**	**(1)**	**(2)**	**(3)**	**(4)**	**(5)**	**(6)**	**(7)**	**(8)**
***(1) PRICE***	1							
***(2) NI***	0.632**	1						
***(3) SALES***	0.611**	0.304**	1					
***(4) BVE***	0.797**	0.555**	0.732**	1				
***(5) TA***	0.802**	0.422**	0.836**	0.834**	1			
***(6) INTAN***	0.487**	0.340**	0.553**	0.514**	0.625**	1		
***(7) CASH***	0.600**	0.420**	0.624**	0.619**	0.675**	0.476**	1	
***(8) DIV***	0.535**	0.600**	0.208**	0.413**	0.393**	0.352**	0.259**	1

Significance:

***0.001,

**0.01,

*0.05.

Pearson, two-tailed.

### Changes in value relevance over time—Basic model

To explore the evolution of value relevance we first run Eq (1) on a yearly basis. Cross data analysis for the basic model (Table 8) shows an increasing trend in value relevance, adjusted R^2^ rising from 0.522 in 2009 to 0.788 in 2016, all coefficients of determination computed for the post-IFRS adoption years being superior to those reported for the years listed companies complied with EU Directives. With one exception, coefficients for net income and book value of equity are both positive and significant, book value of equity being in all cases significant at 0.1%. In 2015, book value of equity acts as a confounder, rendering net income irrelevant, albeit in simple regression analysis, net income is positively correlated with equity prices.

**Table 8:** Evolution in value relevance over time (Basic model).

Years	N	*a*_*1*_	*a*_*2*_	Adj. R^2^	*b*_*1*_	R^2^	*c*_*1*_	R^2^	IncrNI	IncrBVE	CommonNI&BVE
**2009**	57	2.272*	0.294***	0.522	4.353**	0.264	0.352***	0.480	0.042	0.258	0.222
		*(2*.*630)*	*(5*.*684)*		*(4*.*441)*		*(7*.*132)*				
**2010**	58	2.727*	0.335***	0.519	4.946**	0.137	0.361***	0.497	0.022	0.382	0.115
		*(2*.*148)*	*(6*.*870)*		*(2*.*982)*		*(7*.*433)*				
**2011**	55	4.750**	0.486***	0.589	5.642*	0.111	0.501***	0.526	0.063	0.478	0.048
		*(3*.*210)*	*(8*.*046)*		*(2*.*577)*		*(7*.*664)*				
**2012**	56	3.562***	0.306***	0.582	5.686***	0.371	0.402***	0.479	0.103	0.211	0.351
		*(3*.*947)*	*(5*.*447)*		*(5*.*648)*		*(7*.*039)*				
**2014**	61	2.534**	0.343***	0.645	5.912***	0.340	0.410***	0.610	0.035	0.305	0.305
		*(2*.*796)*	*(7*.*312)*		*(5*.*516)*		*(9*.*615)*				
**2015**	61	-0.345	0.728***	0.793	7.827***	0.400	0.712***	0.799	-0.006	0.393	0.406
		*(-0*.*325)*	*(10*.*663)*		*(6*.*222)*		*(15*.*202)*				
**2016**	58	2.711**	0.538***	0.788	7.200***	0.542	0.685***	0.754	0.034	0.246	0.508
		*(3*.*361)*	*(8*.*257)*		*(8*.*148)*		*(13*.*091)*				
**Whole period**	406	2.981***	0.438***	0.687	6.761***	0.399	0.542***	0.634	0.053	0.288	0.346
		*(8*.*364)*	*(19*.*345)*		*(16*.*350)*		*(26*.*448)*				
**2009–2012 EU Directives**	226	2.673***	0.351***	0.532	4.864***	0.188	0.394***	0.485	0.047	0.344	0.141
		*(4*.*962)*	*(12*.*925)*		*(7*.*211)*		*(14*.*516)*				
**2014–2016 IFRS**	180	2.410***	0.504***	0.739	7.096***	0.452	0.608***	0.711	0.028	0.287	0.424
		*(4*.*607)*	*(14*.*078)*		*(12*.*091)*		*(20*.*885)*				

Models:

*P*_*i*_ = *a*_0_ + *a*_1_*NI*_*i*_ + *a*_2_*BVE*_*i*_ + *ε*_*i*_

*P*_*i*_ = *b*_0_ + *b*_1_*NI*_*i*_ + *ε*_*i*_

*P*_*i*_ = *c*_0_ + *c*_1_*BVE*_*i*_ + *ε*_*i*_

Notes: P is equity price three months after year-end. NI is net income, BVE is book value of equity, both scaled by common shares outstanding. IncrNI(BVE) is the incremental explanatory power of net income (book value of equity), while CommonNI&BVE is the combined explanatory power of both variables. t statistic in parenthesis.

Significance:

***0.001,

**0.01,

*0.05.

To analyze further the individual contribution of the components of the basic model we follow Collins *et al*. [49], in decomposing the combined explanatory power of net income and book value of equity into individual and common explanatory power of the two predictors. This strategy assumes that net income and book value of equity “act as substitutes for each other in explaining prices, while they also function as complements by providing explanatory power incremental to one another” [49]. As described by Collins *et al*. [49], the method was first introduced by Theil in 197l and further applied in accounting research, the coefficient of determination of the basic model being decomposed into three components as follows:

Let R_T_^2^ denote the coefficient of determination of the basic model (1), and R_4_^2^ and R_5_^2^ the coefficients of determination of Eq (2) run for earnings and book value of equity:

$$P_{i}=b_{0}+b_{1}{NI}_{i}+\varepsilon_{i}$$ (4)

$$P_{i}=c_{0}+c_{1}{BVE}_{i}+\varepsilon_{i}$$ (5)

The incremental explanatory powers of net income (R_NI_^2^; IncrNI) and book value of equity (R_BVE_^2^; IncrBVE) are determined as: R_NI_^2^ = R_T_2—R_5_^2^ and R_BVE_^2^ = R_T_2 –R_4_^2^. The remaining R_T_^2^—R_NI_^2^—R_BVE_^2^ = R_COM_^2^ denotes the common explanatory power of both net income and book value of equity (R_COM_^2^; CommonNI&BVE).

Table 8 also displays the results of decomposing the total explanatory power of the basic model for BSE.

**Fig 2:**
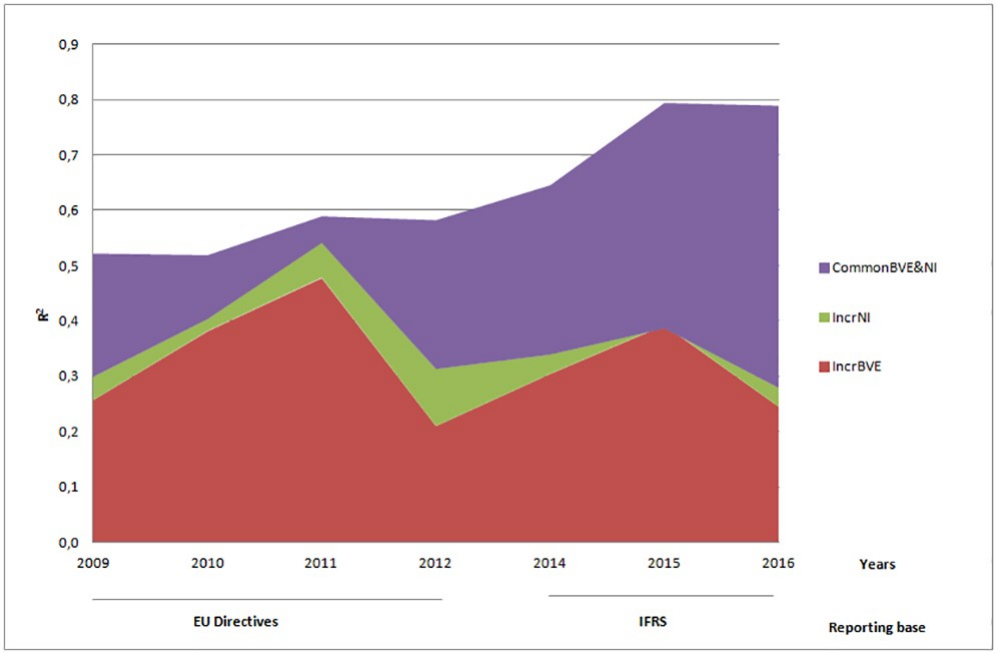
Incremental importance of net income and book value of equity.

**Fig 3:**
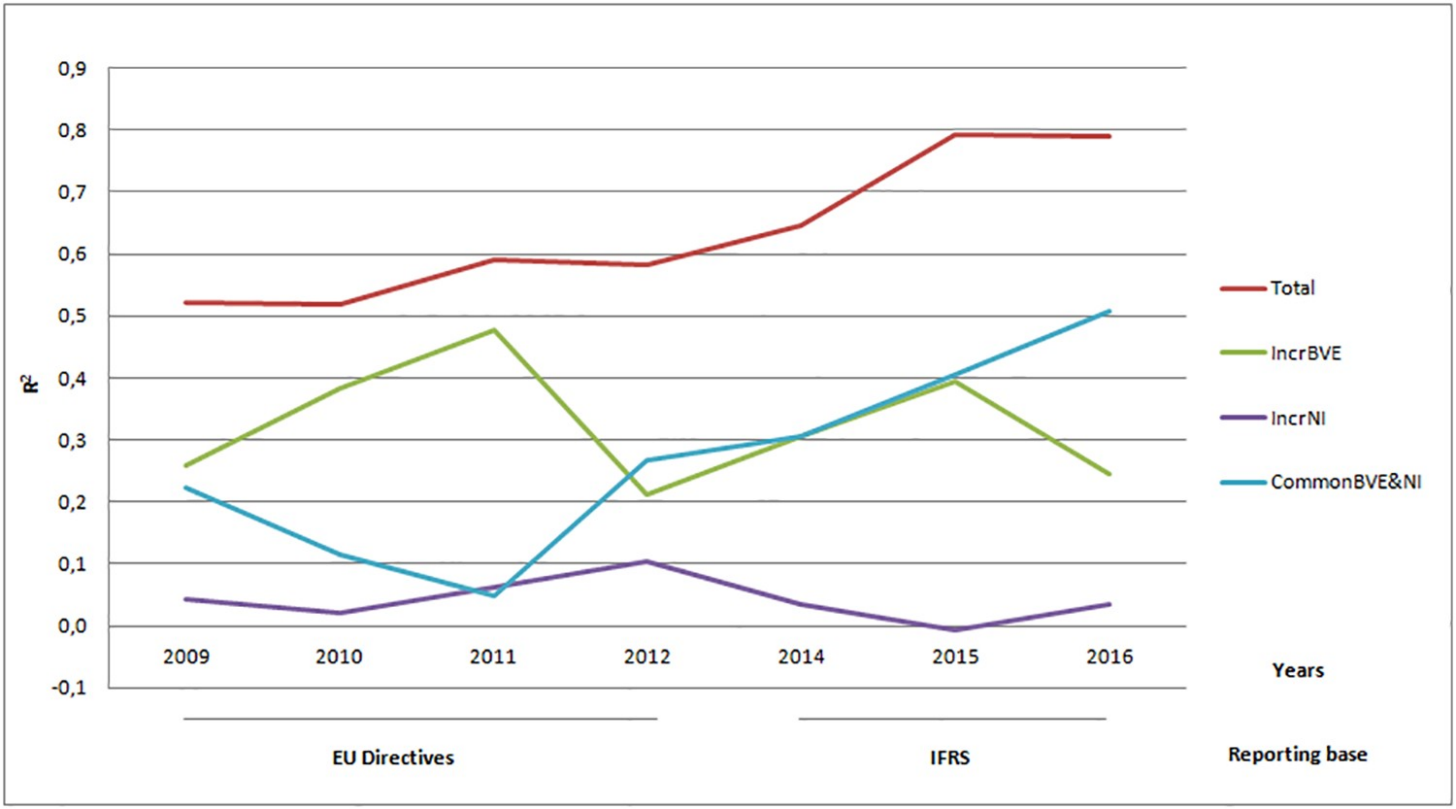
Trends in incremental importance of earnings and book value of equity.

Net income has materially lower incremental explanatory power than book value of equity on the Romanian capital market, with common contribution of the accounting amounts being also substantial. Figs 2 and 3 allow for assessing the magnitude of the Basic Model’s components contribution to the total explanatory power (Fig 2) and their contribution to the growing trend in value relevance (Fig 3).

Fig 2 displays the R^2^ s layered over each other to reach the total explanatory power of the Basic Model. The important incremental contribution of book value of equity seems to give way to the growing contribution of earnings and equity taken together in the post-IFRS adoption years, while the incremental contribution of net income seems to diminish.

Fig 3 displays the trends in the R^2^ s of the components of the Basic Model compared to the trend of its total explanatory power. Observing Fig 3, it appears that the growing trend in value relevance is supported by the uprising tendency of common contribution of net income and book value of equity, while the incremental contribution of each of the two financial indicators seem to slowly decline. The small range of our time series does not allow for testing the significance of the trends.

These results are partially in line with those reported for developed markets, which also show that in recent years, the explanatory power of book value of equity or other balance-sheet variables tends to be superior to that of net income ([47], [48], [49], [53], and report a growing trend of the combined explanatory power of earnings and book values [49]. However, based on recent findings [47], such an overturn in the explanatory power of net income and book value seems to have occurred in turbulent periods of the capital markets, such as the technology bubble in the late 1990s or the financial crisis of 2007–2008. Yet, even in such periods, on developed markets, the explanatory power of net income compared to book value of equity is not as low as the one reported for BSE, which is much closer to the one reported for loss-firms samples [49]. This might be consistent with the characteristics of the Romanian capital market featuring low performing companies, which make investors rely less on earnings and more on book value of equity or both for making investment decisions. Such conjecture is supported by empirical evidence provided for developed markets showing that value relevance of earnings and book value of equity are inversely related to companies’ financial health, book value becoming incrementally more value relevant than earnings as financial health deteriorates [54]. Book value of equity is useful in assessing companies’ ability to generate future economic benefits [55], and it also provides a ‘liquidation value’ in the case of firms in financial distress [54].

### Changes in value relevance—Other financial information

Cross data analysis also reveals that other accounting amounts besides earnings and equity are value relevant (Table 9). Sales, total assets, intangibles, cash holdings and dividends are strongly correlated with market prices (coefficient *b*_*1*_ significant at 0.1% with the exception of intangibles during 2009–2012, significant at 1%).

**Table 9:** Evolution in value relevance over time (Other financial amounts).

**Sales**					
Years	*a*_*0*_	*a*_*1*_	F	N	R^2^
2009	0.330* (2.362)	0.170*** (6.578)	43.275***	57	0.440
2010	0.501** (3.465)	0.313*** (7.582)	57.487***	58	0.507
2011	0.618** (3.458)	0.184*** (5.278)	27.854***	55	0.344
2012	0.360** (2.711)	0.206*** (6.404)	41.012***	56	0.432
2014	0.541** (2.914)	0.284*** (7.693)	59.187***	61	0.501
2015	0.520 (1.860)	0.593*** (8.430)	71.063***	61	0.551
2016	0.289 (1.178)	0.757*** (11.490)	132.025***	58	0.702
*Mean*					0.497
Whole period	0.546*** (6.158)	0.308*** (15.474)	239.458***	406	0.373
2009–2012 EU Directives	0.484*** (6.343)	0.199*** (11.962)	143.099***	226	0.390
2014–2016 IFRS	0.568*** (3.666)	0.473*** (13.117)	172.048***	180	0.493
**Total Assets**					
Years	*a*_*0*_	*a*_*1*_	F	N	R^2^
2009	0.298* (2.051)	0.141*** (6.404)	41.006***	57	0.427
2010	0.488** (2.923)	0.202*** (6.161)	37.954***	58	0.404
2011	0.521** (3.044)	0.127*** (6.222)	38.708***	55	0.422
2012	0.361** (2.705)	0.123*** (6.354)	40.374***	56	0.428
2014	0.385* (2.331)	0.170*** (9.860)	97.211***	61	0.622
2015	0.384 (1.819)	0.242*** (12.658)	160.213***	61	0.734
2016	0.196 (1.512)	0.279*** (24.092)	580.425***	58	0.912
*Mean*					*0*.*564*
Whole period	0.303*** (4.537)	0.207*** (26.995)	728.749***	406	0.644
2009-2012EU Directives	0.444*** (5.728)	0.138*** (12.113)	146.729***	226	0.396
2014–2016 IFRS	0.290** (2.738)	0.237*** (23.735)	563.363***	180	0.761
**Intangibles**					
Years	*a*_*0*_	*a*_*1*_	F	N	R^2^
2009	0.507** (3.033)	42.668** (3.652)	13.333***	57	.195
2010	0.805*** (4.347)	46.330** (3.231)	10.440**	58	.157
2011	0.744** (3.628)	45.559** (3.348)	11.208***	55	.175
2012	0.540** (3.503)	38.464** (3.723)	13.859**	56	.204
2014	0.819** (3.492)	52.145*** (3.993)	15.941***	61	.213
2015	0.981*** (2.511)	108.612*** (4.241)	17.983***	61	.237
2016	0.597 (1.738)	111.610*** (6.552)	42.925***	58	.434
*Mean*					.*231*
Whole period	0.692*** (7.018)	69.395*** (11.192)	125.257***	406	.237
2009-2012EU Directives	0.652*** (7.329)	42.829*** (6.890)	47.467***	226	.175
2014–2016 IFRS	0.812*** (4.343)	88.543*** (8.374)	70.116***	180	.284
**Cash Holdings**					
Years	*a*_*0*_	*a*_*1*_	F	N	R^2^
2009	0.346* (2.316)	4.987*** (5.791)	33.539***	57	0.379
2010	0.717*** (4.107)	3.256*** (4.330)	18.752***	58	0.251
2011	0.701*** (3.764)	1.871*** (4.397)	19.332***	55	0.267
2012	0.423** (3.292)	2.045*** (6.272)	39.338***	56	0.421
2014	0.737** (3.520)	2.263*** (5.467)	29.885***	61	0.336
2015	0.742* (2.504)	3.615*** (7.092)	50.290***	61	0.464
2016	0.593 (1.866)	6.040*** (7.363)	54.210***	58	0.492
*Mean*					*0*.*373*
Whole period	0.642*** (7.419)	3.165*** (15.064)	226.925***	406	0.360
2009-2012EU Directives	0.616*** (7.645)	2.250*** (9.113)	83.052***	226	0.270
2014–2016 IFRS	0.768*** (4.618)	3.540*** (10.628)	112.957***	180	0.390
**Dividends**					
Years	*a*_*0*_	*a*_*1*_	F	N	R^2^
2009	0.505** (3.232)	35.247*** (4.257)	18.122***	57	0.248
2010	0.769*** (4.594)	54.554*** (4.383)	19.215***	58	0.255
2011	0.776*** (4.175)	26.495*** (3.946)	15.571***	55	0.227
2012	0.547*** (3.867)	34.329*** (4.385)	19.228***	56	0.263
2014	0.833*** (3.963)	28.464*** (4.945)	24.450***	61	0.293
2015	1.164** (3.224)	80.778*** (3.782)	14.305***	61	0.198
2016	0.869** (2.960)	22.916*** (7.504)	56.303***	58	0.501
*Mean*					*0*.*284*
Whole period	0.911*** (10.814)	25.316*** (12.728)	161.996***	406	0.287
2009-2012EU Directives	0.676*** (8.297)	32.708*** (8.071)	65.133***	226	0.225
2014–2016 IFRS	1.166*** (7.077)	23.258*** (8.406)	70.668***	180	0.285

Model:

*P*_*i*_ = *a*_0_ + *a*_1_*VAR*_*i*_ + *ε*_*i*_

Notes: P is equity price three months after year-end. VAR is Sales, Total assets, Intangibles, Cash holdings and Dividends, all scaled by common shares outstanding.

t statistic in parenthesis.

Significance:

***0.001,

**0.01,

*0.05.

**Fig 4:**
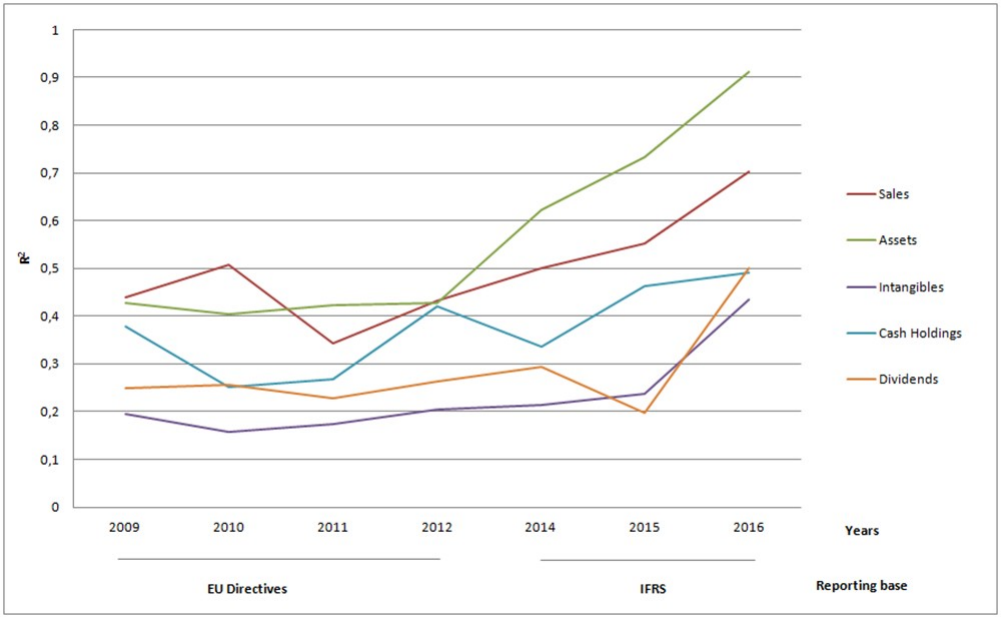
Trends in value relevance over time (Other financial amounts).

Among these variables, the most value relevant are total assets (mean R^2^ 55.4%), sales (mean R^2^ 49.7%) and cash holdings (mean R^2^ 37.3%). Fig 4 shows they also experience growing tendencies, with assets and sales featuring most increasing trends, particularly in the post-IFRS adoption period.

### The impact of IFRS adoption on value relevance

To explore the effect of IFRS adoption in individual financial statements of Romanian listed companies, we first run Eq (1) on pre- and post-adoption pooled samples (results reported in Table 8). For the pre-adoption sample, the value relevance of net income and book value of equity is rather similar to the one reported for developed markets [47], *i*.*e*. for Romania: 53.2% (Adj. R^2^, pooled data 2009–2012) or 55.3% (mean annual Adj. R^2^ for 2009–2012 computed based on data reported in Table 8), compared with 57.7% (mean annual Adj. R^2^ computed for the same period based on data reported by [47]) for the American capital market. Yet, after the adoption of IFRS, there is a substantial increase in value relevance on the Romanian capital market, Adj. R^2^ for post-adoption sample rising to 73.9%. Results are also confirmed by cross data analysis, mean annual Adj. R^2^ for 2014–2016 (computed based on data reported in Table 8) being 74.2%. For 2014, Adj. R^2^ is 64.5% for BSE, while Barth *et al*. [47] report 50.7% for the American stock market.

Increases in value relevance for before and after adoption samples were also observed for all the other financial amounts analysed (pooled data reported in Table 9), the most significant increase being registered for total assets (92.2% increase from 39.6% for the pre-adoption sample to 76.1% computed for the post-adoption sample).

To test the significance of the value relevance increase after the IFRS adoption, we use the Chow test [56], which tests whether the coefficients are equal for regressions run for two subsamples. In Chow’s terms, the method tests whether a given “economic relationship” (in our case, the correlation between accounting information and market prices) “remains stable in two periods of time” [56], the critical event splitting our sample being the IFRS adoption in individual financial statements of Romanian listed companies.

Table 10 reports results for the Chow test, revealing that the increase in value relevance after the IFRS adoption is significant for all the investigated accounting amounts addressed individually, but also for the Basic Model.

**Table 10:** Significance of value relevance increase after the IFRS adoption.

Variable	CHOW Test	
	F	Sig. F
Net income and Book value of equity (*Basic model*)	6.841	0.000
Net income	4.551	0.011
Book value of equity	20.440	0.000
Sales	45.819	0.000
Total assets	28.259	0.000
Intangibles	14.196	0.000
Cash holdings	5.413	0.000
Dividends	4.018	0.019

Given the relative stability of the Romanian capital market during 2009–2016, the significant increase in value relevance could be attributed to the IFRS adoption.

### Relative importance of financial information

In order to investigate the combined contribution of accounting information to stock valuation, we scrutinize Eq (3) in cross-data analysis. We exclude total assets from the multiple regression analysis due to collinearity issues (VIF higher than 10), the informational value of total assets overlapping total sales or book value of equity.

**Fig 5:**
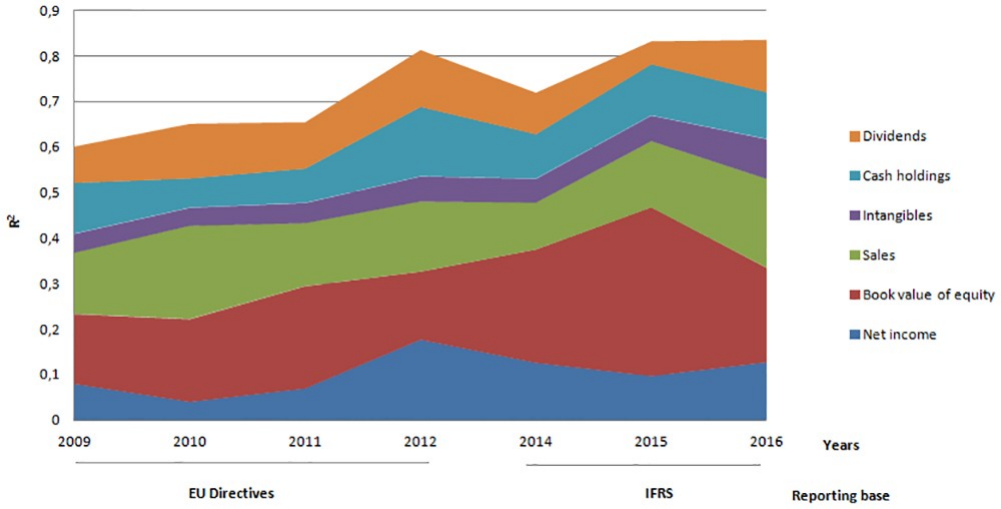
Relative importance of financial information.

To decompose the coefficient of determination, we use *lmg* metric from the package Relaimpo (Relative importance for linear regression in R [52]. Fig 5 shows the decomposed R^2^s strata, showing the contribution of each accounting amount to the total explanatory power of the model (3). As revealed by Fig 5, the most important contribution to the shares valuation seems to be provided by book value of equity, followed by sales for the entire analyzed period. Net income seems far less relevant during 2009–2011, yet, its relative importance features an increasing trend, reaching its maximum in 2012 and relatively maintaining magnitude during post-IFRS adoption years.

A similar perspective on the relative importance of accounting amounts is revealed by pooled data analysis (Table 11), book value of equity ranking first both before and after IFRS adoption. Net income manages to rank second for the entire pooled sample, closely followed by sales, yet, its position is fluctuating when analyzed based on sub-periods. Net income ranks fifth during 2009–2012, after cash holdings and dividends, yet materially improving in the post-adoption years when is ranking third. These findings support our initial assessment of the Basic Model, which revealed the low incremental explanatory power of net income compared to book value of equity on BSE contrary to results reported for developed markets. On the American capital market, Barth *et al*. [47] reports net income ranking first, followed by book value of equity, total assets and sales for their full sample analyzed during 1962–2014.

**Table 11:** Ranks of relative importance of financial information.

Sub-samples	2009–2016		2009–2012		2014–2016	
	Full sample		EU Directives		IFRS	
	Variable importance (%)	Rank	Variable importance (%)	Rank	Variable importance (%)	Rank
Net Income	17.49	(2)	10.86	(5)	15.12	(3)
Book value of equity	34.49	(1)	30.57	(1)	32.56	(1)
Sales	14.43	(3)	22.14	(2)	23.71	(2)
Intangibles	7.24	(6)	6.54	(6)	7.97	(6)
Cash holdings	13.61	(4)	13.68	(4)	11.73	(4)
Dividends	12.74	(5)	16.21	(3)	8.91	(5)
	*100*.*00*		*100*.*00*		*100*.*00*	

These findings are plausible for an emergent market, with low performing companies, where investors are less prone to rely extensively on net income, using other financial information that would allow them to assess companies’ ability to generate future earnings or the net realizable value of companies in financial distress.

To further explore the relative importance of financial information, we analyze R^2^ decomposition for model (3) on sub-samples reflecting the structure of our population, *i*.*e*. loss and profit firms, financial and non-financial companies, as well as companies listed on the Premium or Standard tier.

Table 12 reports total explanatory power of accounting information for sub-samples and the components’ contributions (relative importance summing to 100%), together with the results of the Chow test applied for individual financial amounts revealing whether their value relevance increase significantly after IFRS adoption.

**Table 12:** Ranks of relative importance of financial information.

Sub-samples	**Loss firms**			**Profit firms**		
	Variable importance (%)	Rank	Significant increase after IFRS adoption	Variable importance (%)	Rank	Significant increase after IFRS adoption
Net Income	22.16	(3)	No	23.16	(2)	No
Book value of equity	33.06	(2)	No	29.72	(1)	Yes
Sales	-	-	No	14.06	(3)	Yes
Intangibles	-	-	No	9.93	(5)	Yes
Cash holdings	-	-	No	13.39	(4)	Yes
Dividends	0.12	(4)	No	9.74	(6)	Yes
Total assets	44.66	(1)	No	-	-	Yes
Adj. R^2^	0.483			0.792		
N	108			298		
Sub-samples	**Financials**			**Non-Financials**		
	Variable importance (%)	Rank	Significant increase after IFRS adoption	Variable importance (%)	Rank	Significant increase after IFRS adoption
Net Income	16	(3)	No	16.65	(3)	Yes
Book value of equity	40.2	(1)	No	31.61	(1)	Yes
Sales	19.33	(2)	Yes	15.78	(4)	Yes
Intangibles	10.85	(4)	No	6.29	(6)	Yes
Cash holdings	2.83	(6)	No	17.64	(2)	Yes
Dividends	10.79	(5)	No	12.03	(5)	Yes
Total assets	-	-	Yes	-	-	Yes
Adj. R^2^	0.596			0.757		
N	66			340		
Sub-samples	**Standard tier**			**Premium tier**		
	Variable importance (%)	Rank	Significant increase after IFRS adoption	Variable importance (%)	Rank	Significant increase after IFRS adoption
Net Income	12.60	(4)	No	20.28	(2)	Yes
Book value of equity	40.82	(1)	Yes	31.22	(1)	Yes
Sales	21.41	(2)	Yes	14.75	(3)	Yes
Intangibles	2.58	(6)	No	11.34	(4)	Yes
Cash holdings	17.13	(3)	No	11.10	(6)	Yes
Dividends	5.46	(5)	No	11.31	(5)	Yes
Total assets	-	-	Yes	-	-	Yes
Adj. R^2^	0.610			0.815		
N	285			121		

The highest value relevance and the largest impact of IFRS on value relevance is registered for companies listed on the Premium tier and for profit-firms, which is to be expected as, being the performers of the market, these companies are presumably more scrutinized by investors. Financial information explains 81.5% of the variation in market prices of Premium shares, with all accounting amounts featuring significant increases after IFRS adoption. For profit-firms, financial information explains 79.2% of equity prices, significant increases in value relevance being registered for all accounting amounts except net income.

On the opposite side, the value relevance for loss-firms is the lowest (only 48.3%). In the case of loss firms, we found the same baffling significant negative correlation between net income and equity prices, which was previously reported on the American stock market [57], [58], and was explained as due to the model misspecifications. Barth and Kallapur [57] suggest controlling for scale effects, as big companies have bigger share prices and tend to incur higher losses, inducing a negative bias to earnings’ coefficient. Although book value of equity is proposed as one of the proxies for size that could normalize the earnings coefficient [57], Collins *et al*. [58] provide evidence that in the case of loss-firms, book value of equity performs its already established functions of providing information on the companies’ future normal earnings or its liquidation value. In our case, adding book value of equity to net income does not remove the significance of the correlation, yet total assets does, with the earnings’ coefficient remaining still negative. The only variable that could also be included in the model without featuring abnormal significant negative correlations was dividends per share. Total assets prove to be the most relevant for loss-firms, which does not confound book value of equity in terms of relative importance, suggesting those variables playing different roles in stock valuation.

IFRS adoption has no significant impact on the relevance of financial information reported by loss-firms. IFRS adoption has also no significant impact on the value relevance of accounting amounts reported by financial companies, with the exception of sales, which could be explained by the relative constancy in the transparency and quality of financial disclosure of these entities both before and after IFRS adoption. Another explanation could be the fact that banks already prepared IFRS compliant individual financial statements as a mandatory second set of accounts since 2009. This is obviously not the case for non-financial companies, for which the relevance of all financial amounts increased significantly in the post-adoption years.

In terms of relative importance, book value of equity ranks first for all sub-samples except loss-firms, with net income ranking second for better performing companies (profit-firms and Premium tier companies), being the closest to book value of equity in the case of profit-firms (6.56% difference in terms of relative importance). Sales seem to matter more for financial entities and Standard tier shares, while cash holdings and dividends seem to count more for non-financial companies.

### Conclusions

The paper examined the changes is value relevance of financial information over time for an emergent capital market, showing that after the IFRS adoption in individual financial statements, accounting amounts become more relevant for stock market valuations. We used regression analysis and decomposition techniques for the total explanatory power of the models used in order to assess the magnitude of value relevance of financial indicators, as well as their relative importance. Following Barth *et al*. [47], we explored not only accounting amounts traditionally addressed by the literature (net income and book value of equity), but also other financial information that is expected to play a role in explaining equity prices (sales, total assets, intangibles, cash holdings, and dividends).

We find that all financial amounts investigated are significantly correlated with equity prices, and that their value relevance features increasing trends. Although increases in value relevance vary among the accounting variables analyzed, our results show that increases are significant after IFRS adoption in all cases.

In terms of relative importance, our findings show that book value of equity is the most relevant accounting amount, while net income ranks second, being even outranked by other financial data (*e*.*g*. sales) in cross-data analysis, particularly for the beginning of our analyzed period. These results are found to be at odds with those reported for developed markets, where net income is the most value relevant, and are explained by the low performance of the Romanian listed companies, which drives investors to rely less on earnings and more on indicators that can help assess the companies’ ability to generate future earnings, or provide a proxy for liquidation value.

Our findings also suggest that relative importance of different financial information varies for different categories of companies investigated, which is also the case for the impact of IFRS adoption. We find that IFRS adoption had a significant impact on value relevance for performers of the Romanian market (Premium tier companies and profit-firms) but not for loss-firms. We also find that IFRS adoption did not significantly increased value relevance for financial entities.

The main limitation of the paper comes from the relatively small number of observation compared to studies conducted on established markets, which is further amplified by missing data. However, as argued elsewhere [24], this is a commonality for studies dealing with developing markets, particularly for post-communist countries, where markets are young and the number of listed companies is small.

This paper contributes to the literature by providing empirical evidence of the benefits of IFRS adoption on a developing market, showing that the IFRS adoption in individual accounts of listed companies led to an increase in value relevance. The paper also provides a more comprehensive perspective on the value relevance of a broader array of financial amounts, which is rarely done for emerging markets. Our results suggest that net income might not be the most relevant financial information on emergent markets, which may advise future research in this area to avoid focusing only on earnings, and also address other accounting amounts when exploring value relevance. An interesting development for future research on emergent markets could be providing empirical evidence on the actual roles played by different financial variables in stock valuations, and analyze the impact of IFRS adoption in the context of other drivers of value relevance.
